# Supplementary material for: Apoptotic Cell Death in Bicuspid-Aortic-Valve-Associated Aortopathy
Source: Int J Mol Sci. 2023 Apr 18;24(8):7429. doi: 10.3390/ijms24087429 (PMC10138609; doi:10.3390/ijms24087429)
Supplement: Supplementary file 1 [file ijms-24-07429-s001.zip › ijms-2285688-supplementary.pdf]

Supplementary materials

Primary antibodies

Caspase-3 (polyclonal, rabbit), 1:1000	Cell Signaling Technology (#9662), Frankfurt am Main, Germany
mTOR (7C10) (monoclonal, rabbit), 1:1000	Cell Signaling Technology (#2983), Frankfurt am Main, Germany
Akt (pan) (40D4) (monoclonal, mouse), 1:2000	Cell Signaling Technology (#2920), Frankfurt am Main, Germany
ERK (16 ERK/ pan) (monoclonal, mouse), 1:4000	BD Transduction Laboratories™ (#610123), Heidelberg, Germany
Bcl-2 (124) (monoclonal, mouse), 1:1000	Cell Signaling Technology (#15071), Frankfurt am Main, Germany
Vimentin (V9) (monoclonal, mouse), 1:200	Sigma-Aldrich(#V6630), Steinheim, Germany
α-Smooth Muscle Actin (D4K9N) XP® (monoclonal, rabbit), 1:2000	Cell Signaling Technology (#19245), Frankfurt am Main, Germany
p62 (polyclonal, guinea pig), 1:1000	Progen (#GP62-C), Heidelberg, Germany
LC3A (polyclonal, rabbit), 1:300	Novus Biologicals (#NB100-2331), Wiesbaden, Germany
AGE (polyclonal, rabbit), 1:10000	Abcam (#ab23722), Berlin, Germany
RAGE (polyclonal, rabbit), 1:1000	Abcam (#ab37647), Berlin, Germany
GAPDH (polyclonal, rabbit), 1:4000	Abcam (#ab9485), Berlin, Germany
α-Tubulin (B-5-1-2) (monoclonal, mouse), 1:4000	Sigma-Aldrich (#T6074), Steinheim, Germany
β-Actin (AC-15) (monoclonal, mouse), 1:5000	Sigma-Aldrich (#A5441), Steinheim, Germany

Secondary antibodies

Goat anti-rabbit IgG, HRP-conjugated, 1:8000	Cell Signaling Technology (#7074), Frankfurt am Main, Germany
Rabbit anti-mouse IgG, HRP-conjugated,1:8000	Sigma-Aldrich (#A9044), Steinheim, Germany
Rabbit anti-guinea pig IgG, HRP-conjugated, 1:8000	Abcam (#ab6771), Berlin, Germany

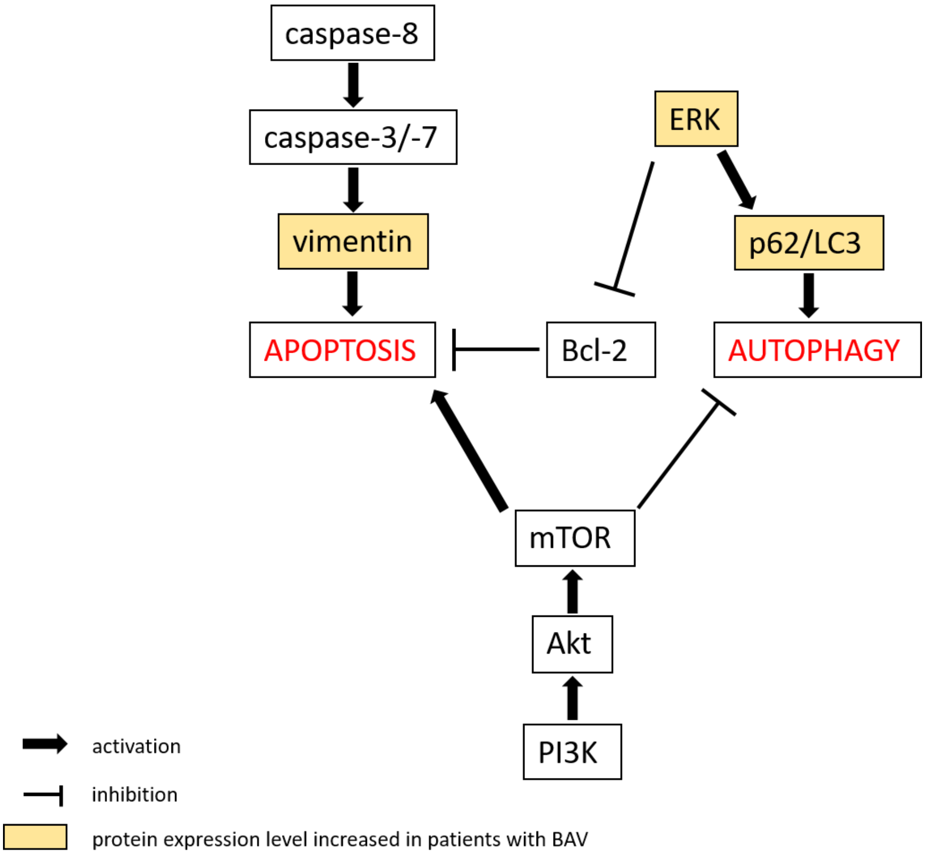

Supplementary Figure S1: Potential pathway and crosstalk between apoptosis and autophagy. Apoptosis is induced by the activation of pro-apoptotic proteins like caspase-8, which in turn activates caspase-3/-7 and vimentin. It can be inhibited by the anti-apoptotic protein Bcl-2. The PI3K/Akt/mTOR pathway also contributes to the induction of apoptosis and to the inhibition of autophagy. In contrast, the interaction of p62, regulated by ERK, and LC3 leads to the activation of autophagy. ERK also inhibits the pro-apoptotic protein Bcl-2. The relative protein expression levels of vimentin, ERK and p62 were increased in tissues of patients with bicuspid aortic valve (BAV).
